# Circulating Follicular Helper and Follicular Regulatory T Cells Are Severely Compromised in Human CD40 Deficiency: A Case Report

**DOI:** 10.3389/fimmu.2018.01761

**Published:** 2018-08-06

**Authors:** Maria Pia Cicalese, Jolanda Gerosa, Manuela Baronio, Davide Montin, Francesco Licciardi, Annarosa Soresina, Rosa Maria Dellepiane, Maurizio Miano, Lucia Augusta Baselli, Stefano Volpi, Carlo Dufour, Alessandro Plebani, Alessandro Aiuti, Vassilios Lougaris, Georgia Fousteri

**Affiliations:** ^1^San Raffaele Telethon Institute for Gene Therapy (HSR-TIGET), IRCCS San Raffaele Scientific Institute, Milan, Italy; ^2^Pediatric Immunohematology and Bone Marrow Transplantation Unit, IRCCS San Raffaele Scientific Institute, Milan, Italy; ^3^Vita-Salute San Raffaele University, Milan, Italy; ^4^Division of Immunology Transplantation and Infectious Diseases (DITID), Diabetes Research Institute (DRI) IRCCS San Raffaele Scientific Institute, Milan, Italy; ^5^Department of Clinical and Experimental Sciences, Pediatrics Clinic and Institute of Molecular Medicine A. Novicelli, University of Brescia, ASST-Spedali Civili of Brescia, Brescia, Italy; ^6^Immuno-Rheumatology, Department of Paediatrics II, Regina Margherita Hospital, Città della Salute e della Scienza di Torino, Torino, Italy; ^7^Pediatrics Clinic, ASST-Spedali Civili di Brescia, Brescia, Italy; ^8^Department of Pediatrics, Fondazione IRCCS Cà Granda Ospedale Maggiore Policlinico, University of Milan, Milan, Italy; ^9^Department of Emato-Oncology, IRCCS Instituto Giannina Gaslini, Genoa, Italy; ^10^Department of Pediatrics, IRCCS Instituto Giannina Gaslini, Genoa, Italy

**Keywords:** hyper-IgM syndrome, follicular helper T cells, follicular regulatory T cells, class switch recombination, somatic hypermutation, *AICDA*, *CD40LG*, *CD40*

## Abstract

Mutations in genes that control class switch recombination and somatic hypermutation during the germinal center (GC) response can cause diverse immune dysfunctions. In particular, mutations in *CD40LG, CD40, AICDA*, or *UNG* cause hyper-IgM (HIGM) syndrome, a heterogeneous group of primary immunodeficiencies. Follicular helper (Tfh) and follicular regulatory (Tfr) T cells play a key role in the formation and regulation of GCs, but their role in HIGM pathogenesis is still limited. Here, we found that compared to CD40 ligand (CD40L)- and activation-induced cytidine deaminase (AICDA)-deficient patients, circulating Tfh and Tfr cells were severely compromised in terms of frequency and activation phenotype in a child with CD40 deficiency. These findings offer useful insight for human Tfh biology, with potential implications for understanding the molecular basis of HIGM syndrome caused by mutations in *CD40*.

## Background

Hyper-IgM (HIGM) syndromes comprise a group of rare primary immunodeficiencies characterized by low or absent IgG and IgA and normal to elevated levels of IgM (1, 2). CD40 ligand (CD40L) deficiency is X-linked and the estimated frequency is 2:1,000,000 males (3). Although no data are available on the frequency of activation-induced cytidine deaminase (AID) deficiency, this disorder is estimated to affect less than 1:1,000,000 individuals (4). In contrast, there are only a few reported cases of CD40 and uracil *N*-glycosylase (UNG) deficiencies (5–9).

Patients affected by HIGM present defects in class switch recombination and somatic hypermutation, two events that take place in germinal centers (GCs) (2–6). These processes are compromised in HIGM patients due to impaired cross-talk between T and B cells caused by mutations in *CD40LG* and *CD40* or intrinsic B cell defects due to mutations in *AICDA* and *UNG* (10, 11). Clinically, affected patients present recurrent respiratory and gastrointestinal infections, and in some cases autoimmune manifestations (1–3). Patients with mutations in *CD40L* or *CD40* are particularly susceptible to *Pneumocystis carinii* pneumonia (PCP) and *Cryptosporidium* infection (3, 7). Autoimmune manifestations may occur in all forms of HIGM, although they seem more frequent in AID deficiency (12, 13). Two recent studies reported a reduction in the percentage of CD4^+^CD25^+^FOXP3^+^ T regulatory cells (Tregs), accompanied by an increase in the Th17/Treg cell ratio and Th1/Treg cell ratio in *CD40L*-deficient patients (14, 15). Similarly, patients with mutations in *AICDA*, but not UNG-deficient patients, displayed Treg cells with defective suppressive function (16). These results may explain the increased susceptibility to autoimmunity in these disorders.

Follicular helper T cells (CXCR5^+^FOXP3^−^) (Tfh) and follicular regulatory T cells (CXCR5^+^FOXP3^+^) (Tfr) are considered key players for the formation and regulation of GCs, respectively (17–19). To date, available data regarding these T cell subsets in HIGM syndromes are limited and in some cases discordant (16, 20, 21). Furthermore, studies on Tfh and Tfr cells in humans with an extremely rare form of HIGM caused by CD40 deficiency are completely lacking. Here, we report for the first time the distribution and phenotype of circulating Tfh and Tfr cells in a patient with CD40 deficiency and compare them to CD40L and AID-deficient patients.

## Methods

### Clinical Cases and Controls

Ten patients with diagnosis of monogenic HIGM syndromes referred from the Pediatrics Clinic, University of Brescia and ASST-Spedali Civili of Brescia, Fondazione IRCCS Cà Granda Ospedale Maggiore Policlinico, Milan, IRCCS Instituto Giannina Gaslini, Genoa and Immuno-rheumatology, II, Regina Margherita Hospital, Turin, Italy, were enrolled in the study. All the subjects, their parents or guardians provided a written informed consent. The study was approved by the local ethics committee (Comitato Etico Ospedale San Raffaele, Milano) and performed in accordance with the Declaration of Helsinki.

The study group included one pediatric patient with CD40 deficiency, three patients with AID deficiency (one adult and two pediatric) and six patients with CD40L deficiency [two adult and four pediatric (one patient was analyzed in pediatric and adult age)]. Clinical characteristics of the study participants are shown in Table 1. Seven pediatric (6 months–10 years) and nine adult (23–40 years) healthy donors were included as controls (HC).

**Table 1:** Patients’ mutations, clinical characteristics and immunological profile.

Patient	Age (years)	Sex	Genetic defect	Mutation	CD3^**+**^ cells/μl (nv) (34)	CD3^**+**^CD4^**+**^ cells/μl (nv) (34)	CD3^**+**^CD8^**+**^ cells/μl (nv) (34)	CD19^**+**^ cells/μl (nv) (34)	CD16^**+**^CD56^**+**^ cells/μl (nv) (34)	IgG g/l (nv) (35)	IgA g/l (nv) (35)	IgM g/l (nv) (35)	Clinical manifestations	Treatment
Pt. 1	15	F	*CD40*	c. 408A>T EX5 skip^b^	2,000 (1,000–2,000)	1,507 (400–2,000)	356 (200–800)	438 (200–600)	302 (100–700)	**1.80** (6.4–19)	**<0.06** (0.6–3)	0.80 (0.6–3)	*Pneumocystis* infection Cryptosporidium infection Recurrent respiratory infections Liver insufficiency Exitus	IVIG, TMP–SMZ

Pt. 2	31	M	*AICDA*	c.441C>A^b^; p.C147X;	**6,267** (600–2,000)	908 (400–1,200)	**4,092** (200–800)	136 (100–500)	348 (100–500)	**<0.35** (6.4–19)	**<0.06** (0.6–3)	**43.90** (0.6–3)	Recurrent respiratory infections Bronchiectasis Splenomegaly	SCIG
Pt. 3	3	F	*AICDA*	c.389A>C^b^; p.H130P;	2,735 (1,200–4,000)	1,006 (600–2,200)	1,172 (400–1,400)	480 (300–1,500)	**914** (100–800)	**<0.6** (4.6–17)	**<0.02** (0.3–1.7)	**6.80** (0.6–2.6)	Recurrent respiratory infections	IVIG
Pt. 4	14	F	*AICDA*	c.70C>T^b^ p.R24W	na	na	na	na	na	na	na	na	Recurrent respiratory infections	na

Pt. 5	14/19^a^	M	*CD40LG*	c.346+4G>C	**2,264** (800–1,800)	**1,485** (400–1,200)	636 (200–800)	**813** (100–500)	177 (100–700)	**0.18** (6.4–19)	**0.02** (0.6–3)	1.56 (0.6–3)	na	na
Pt. 6	4	M	*CD40LG*	c.487G>T; p.V163F	3,490 (1,200–4,000)	**2,825** (600–2,200)	537 (400–1,400)	1,056 (300–1,500)	251 (100–800)	**<0.6** (5.3–19.6)	**<0.02** (0.4–2.6)	2.60 (0.5–3)	Recurrent respiratory infections	IVIG, AZM
Pt. 7	3	M	*CD40LG*	p.T254P	na	na	na	na	na	na	na	na	Neutropenia Recurrent skin infection (impetigo)	na
Pt. 8	7	M	*CD40LG*	c.761C>T; p.T254M	**4,275** (800–3,200)	**3,198** (400–2,000)	844 (400–1,400)	791 (200–1,000)	192 (100–700)	**<0.45** (6.3–10)	**<0.04** (0.4–3)	**3.75** (0.6–2.6)	Recurrent media otitis Hematuria by *Proteus mirabilis*	IVIG, TMP–SMZ
Pt. 9	30	M	*CD40LG*	p.C682T	na	Na	na	na	na	na	na	na	Neutropenia Aphthous stomatitis Warts	na
Pt. 10	2	M	*CD40LG*	c.585dupA; p.L195fs	na	na	na	na	na	**3.25** (4.6–17)	**<0.07** (0.17–1.8)	1.22 (0.6–2.6)	*Candida* esophagitis Perianal abscess, ileocecal fistula, and multiple colic ulcers with perforation	BMT

*IVIG, intravenous immunoglobulin; TMP–SMZ, trimethoprim/sulfamethoxazole; SCIG, subcutaneous immunoglobulin; AZM, azithromycin; BMT, bone marrow transplantation; na, data not available; nv, normal values*.

*In bold the abnormal values*.

*^a^Pt.5 was analyzed twice, as a pediatric patient (14 years old) and as an adult patient (19 years old)*.

*^b^Homozygous mutation*.

### Sample Collection and Analysis

Peripheral blood mononuclear cells (PBMCs) were isolated by density-gradient centrifugation over Lymphoprep (Stemcell) from heparinized venous blood and frozen in RPMI (Lonza) with addition of 10% DMSO and 25% FBS. PBMCs were then thawed in water bath at 37° and stained for flow cytometry with superficial antibodies against CD4 (SK3), CD3 (SK7), CD45RA (H100), CD19 (4G7), CD14 (TUK4), CD8 (BW135/80), CXCR5 (RF8B2), PD-1 (eBioJ105), and ICOS (ISA-3) (Ab clones are indicated in the parentheses). Cells were fixed and permeabilized for intracellular staining with the FOXP3 Transcription Factor Staining Buffer Set (eBioscience) and stained with antibodies against FOXP3 (259D). Cells were acquired on FACSCantoII (BD) and analyzed with FlowJo (Tree Star) software. Mann–Whitney statistical analysis was performed between HC and patient groups with the prism software (San Diego).

## Results

### Summary of Case Report

The CD40 deficient patient was born from first-degree consanguineous Italian parents. The patient was admitted at the age of 4 months for severe respiratory distress due to PCP, and subsequently re-admitted at the age of 2 years for another pneumonia event. During the second infectious episode, immunological work-up showed low IgG and IgA with normal IgM serum levels and received immunoglobulin replacement treatment and trimethoprim/sulfamethoxazole prophylaxis (Table 1). During follow-up, the patient presented recurrent respiratory infections. The clinical history was complicated by *Cryptosporidium* infection at the age of 12 years that led to progressive end-stage liver dysfunction. The patient over time developed esophageal varices, diffuse abdominal spider naevi with ascitis and severe mucositis and became transfusion dependent. Considering the patient’s severe clinical course, the parents decided against hematopoietic stem cell transplantation. The severity of her clinical conditions led to exitus at the age of 16 years (5).

### Tfh and Tfr Cell Frequencies in Human CD40 Deficiency

**Figure 1:**
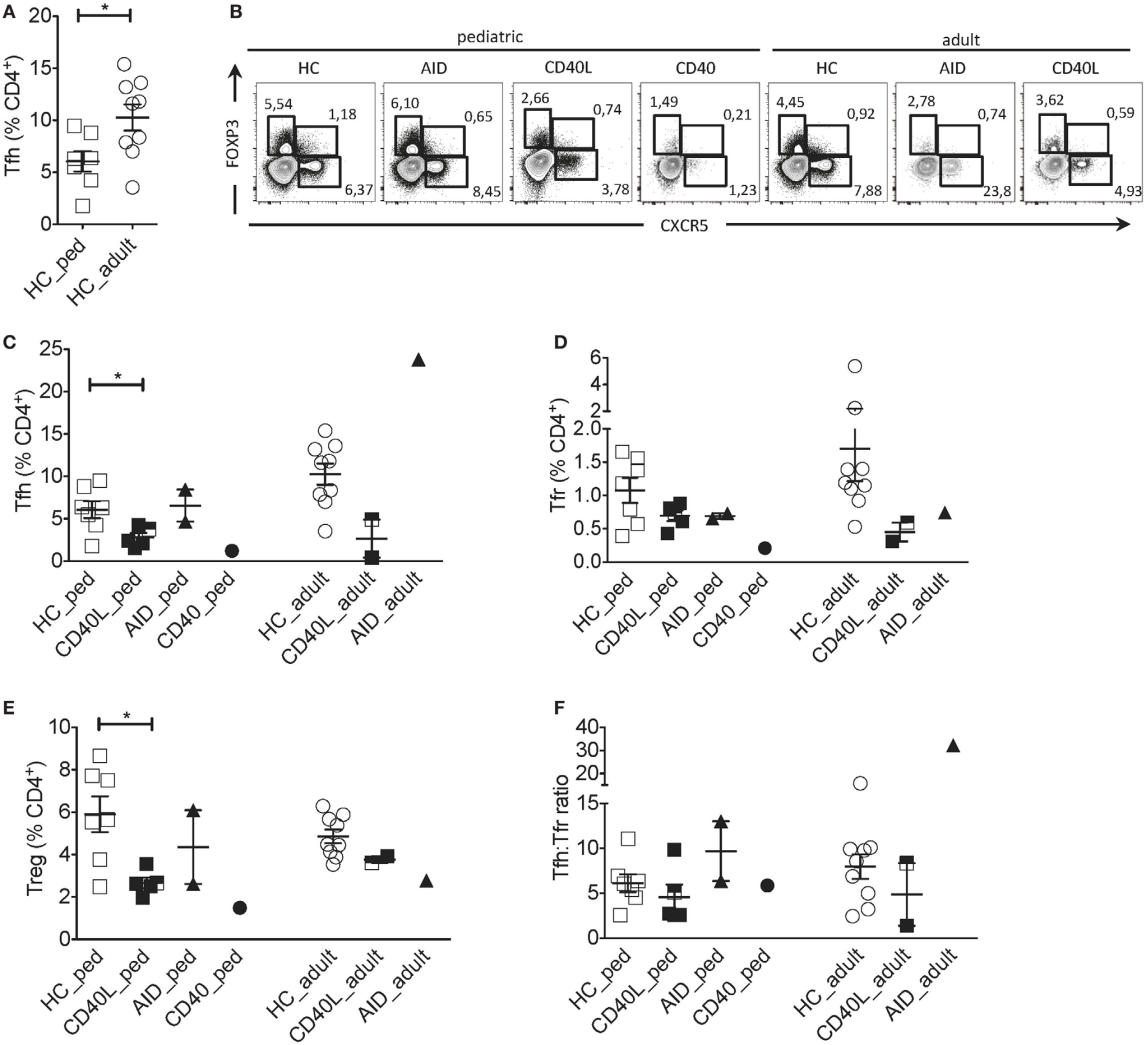
Circulating Tfh and Tfr cells in an individual with CD40 deficiency compared to patients with *CD40L* and *AICDA* mutations. **(A)** Percentage of Tfh (gated on singlets → lymphocytes → CD3^+^CD19^−^CD14^−^CD8^−^ → CD4^+^CD3^+^) in healthy pediatric (3–15 years, *n* = 7, HC_ped) and healthy adult (19–31 years, *n* = 9; HC_adult) healthy controls (HCs). **(B)** Representative flow cytometry plots for Tfh (CXCR5^+^FOXP3^−^), Tfr (CXCR5^+^FOXP3^+^), and T regulatory (Treg) (CXCR5^−^FOXP3^+^) cells, gated on singlets → lymphocytes → CD3^+^CD19^−^CD14^−^CD8^−^ → CD4^+^CD3^+^ in hyper-IgM (HIGM) patients and age-matched HCs. **(C–E)** Percentage of Tfh **(C)**, Tfr **(D)**, and Treg **(E)** cells in pediatric [CD40 ligand (CD40L), *n* = 5; activation-induced cytidine deaminase (AID), *n* = 2; CD40, *n* = 1] and adult (CD40L, *n* = 2; AID, *n* = 1) HIGM patients, compared with age-matched HCs [same as in panel **(A)**]. **(F)** Tfh:Tfr ratio cells in pediatric and adult HIGM patients (AID, CD40L, and CD40 deficiency, as indicated) compared with age-matched HCs. Bars: mean ± SEM. *p < 0.05 (Mann–Whitney test). Each dot represents one patient. Black & white squares represent longitudinal measurements of the same CD40L-deficient patient, collected at 14 and 19 years of age.

Comparison between pediatric and adult HCs showed differences in the percentage of Tfh cells: pediatric HCs had lower frequency of Tfh cells within CD4^+^ T cells when compared with adult HCs (Figures 1A,B), reflecting the expansion of this memory T cell subset with age. Instead, frequencies of Tfr and Treg (CXCR5^−^FOXP3^+^) cells did not change with age (not shown). Since Tfh cells differed significantly between pediatric and adult HCs, patients were compared with age-matched HCs. Tfh, Tfr, and Treg cells were severely reduced in the child with CD40 deficiency as compared with healthy children (Figures 1B–E). Reduction in Tfh and Treg but not in Tfr cell frequencies were also observed in children with CD40L deficiency as compared with age-matched HCs, while children with AID deficiency had normal distribution of all cell subsets (Figures 1B–E).

The circulating Tfh:Tfr ratio provides important insights into the function and regulation of GC responses when evaluated alongside the frequency of Tfh and Tfr. For example, an increased ratio of Tfh:Tfr was found in patients with Sjogren’s syndrome (22), suggesting the persistence of ongoing GC reactions. We observed that despite the alterations in Tfh and Tfr cells in CD40, CD40L and AID deficient pediatric patients, the ratio of Tfh:Tfr was similar to that observed in age-matched HCs (Figure 1F).

Circulating Tfh, Tfr and Treg cells were also reduced in adult CD40L-deficient patients compared with age-matched HCs (Figures 1B–E). While the Tfh:Tfr ratio was conserved in one adult CD40L-deficient patient, it was reduced in the other one (Figure 1F). PBMCs from one CD40L-deficient patient were examined at a pediatric and an adult age (marked with bicolor). The percentage of Tfh and Tfr cells remained relatively stable during the follow-up (Figure 1C). On the contrary, Tfh cell frequency was increased in the single adult AID-deficient patient (Figure 1C), while Tfr and Treg cells were not affected (Figures 1D,E).

### Activation Phenotype of Tfh and Tfr Cells in Human CD40 Deficiency

**Figure 2:**
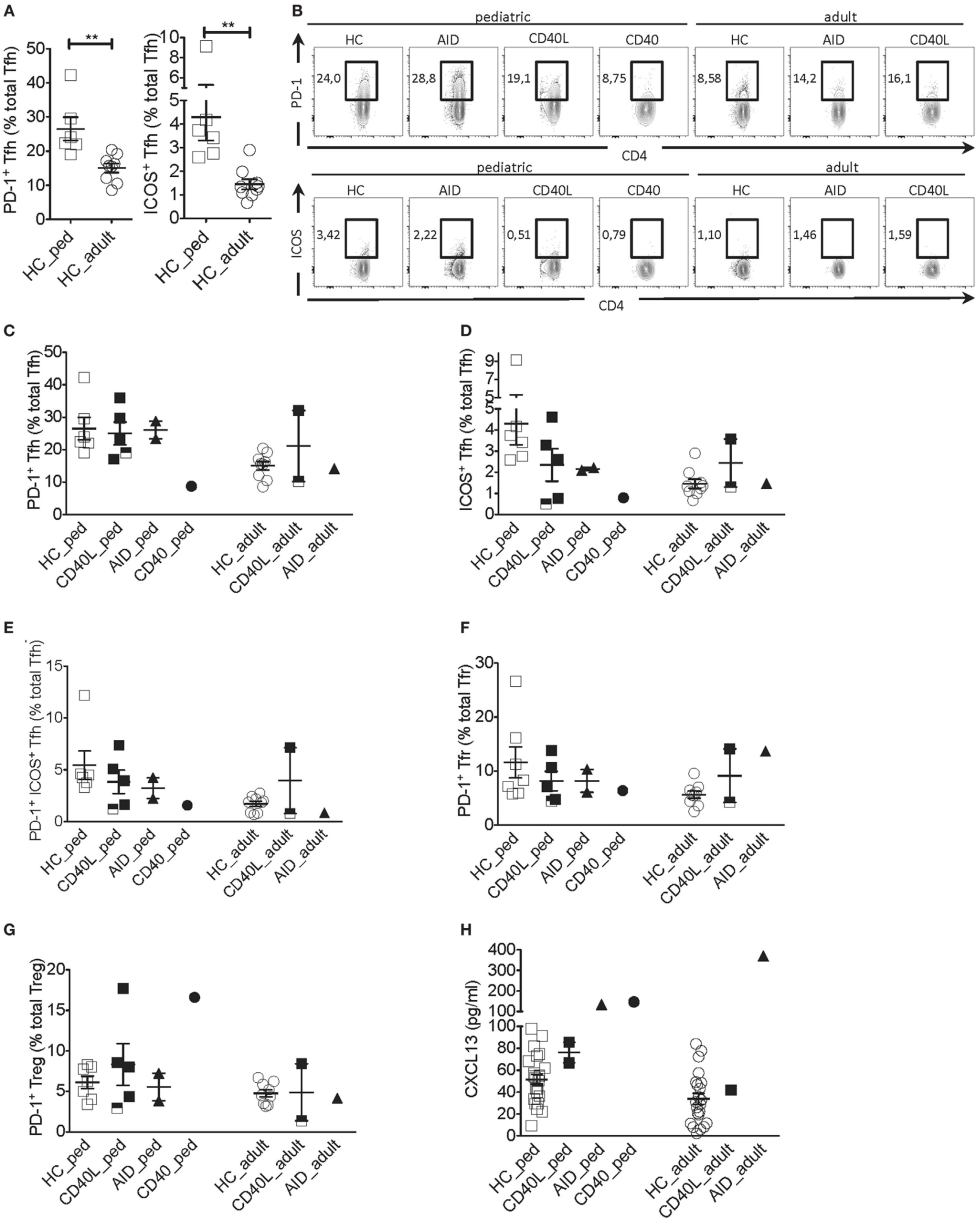
Tfh cell activation phenotype in a patient with CD40 deficiency compared with patients with *CD40L* and *AICDA* mutations. **(A)** Percentage of PD-1^+^ and ICOS^+^ Tfh cells in peripheral blood of a pediatric (HC_ped) and adult (HC_adult) healthy controls (HCs) as described in Figure 1 (gated on singlets → lymphocytes → CD3^+^CD19^−^CD14^−^CD8^−^ → CD4^+^CD3^+^ → CD4^+^CXCR5^+^). **(B)** Representative plots for PD-1^+^ and ICOS^+^ within Tfh cells in hyper-IgM (HIGM) patients and age-matched HCs. **(C,D)** Percentage of PD-1^+^ **(C)** and ICOS^+^ **(D)** Tfh cells in pediatric and adult HIGM patients and age-matched HCs (same as in Figure 1). **(E)** Percentage of PD-1^+^ICOS^+^ Tfh cells in pediatric and adult HIGM patients compared with age-matched HCs. **(F,G)** The proportion of Tfr **(F)** and T regulatory (Treg) **(G)** cells expressing PD-1 in pediatric and adult HIGM patients compared with age-matched HCs. **(H)** CXCL13 levels in the plasma of pediatric [CD40 ligand (CD40L), *n* = 2; activation-induced cytidine deaminase (AID), *n* = 1; CD40, *n* = 1] and adult HIGM patients (CD40L, *n* = 1; AID, *n* = 1) and age-matched HCs (pediatric HC, *n* = 25, adult HC *n* = 23). Bars: mean ± SEM. **p < 0.01 (Mann–Whitney test). Each dot represents one patient. Black & white squares represent longitudinal measurements of the same CD40L-deficient patient, collected at 14 and 19 years of age.

Next, we addressed the expression of PD-1 and ICOS, two molecules typically expressed by activated Tfh cells that correlate with Tfh cell function (17–19). Given that the proportion of Tfh cells expressing PD-1 and ICOS was higher in pediatric as compared with adult HCs (Figures 2A,B), clinical samples were compared with age-matched HCs. The proportion of PD-1^+^ Tfh cells but not the PD-1 geometric mean fluorescence intensity (gMFI) was severely reduced in the CD40-deficient child but not the CD40L and AID-deficient children when compared with HCs (Figures 2B,C; Figure S1A in Supplementary Material). The proportions of ICOS^+^ and PD-1^+^ICOS^+^ Tfh cells but not ICOS gMFI were reduced in several patients affected by HIGM when compared with HCs, irrespective the genetic defect. However, the reduction in ICOS was more pronounced in the CD40-deficient child and some patients with CD40L deficiency (Figures 2B,D,E and data not shown).

Interestingly, the proportion Tfr and Treg cells expressing PD-1 was elevated in the pediatric CD40-deficient patient (Figure 2G). Treg cells also expressed higher levels of PD-1 in this patient (Figure S1C in Supplementary Material). Instead, the proportion and expression levels of PD-1 on Tfr and Treg cells in children with CD40L and AID deficiency were similar to those seen in HCs (Figures 2F,G; Figures S1B,C in Supplementary Material). The percentage of PD-1^+^ Tfr cells was slightly elevated in one adult CD40L-deficient and the adult AID-deficient patient as compared with HCs (Figures 2F,G).

Unexpectedly, plasma levels of CXCL13, a chemokine considered to be indicative of GC activity (23), were found elevated in the patient with CD40-deficiency, but were within the normal range in the CD40L-deficient patients (Figure 2H). Patients with AID deficiency had also elevated plasma levels of CXCL13 compared with HCs, in line with the hyper-reactive GCs described in this disorder (17).

## Discussion

Our findings report the first phenotypic characterization of Tfh and Tfr in human CD40 deficiency and describe differences to other HIGM syndromes caused by mutations in *CD40L* and *AICDA*. The single patient with CD40 deficiency analyzed, showed a marked reduction in circulating Tfh and Tfr cells suggesting that lack of CD40 signals contribute to an early demise of GCs in humans, similarly to mice (24). While the precise mechanism of CD40 signaling is poorly understood, CD40 ligands on T cells and follicular dendritic cells (FDCs) were shown to be essential for T-dependent and T-independent GC responses, respectively (25). In contrast to other forms of HIGM and particularly the CD40L deficiency, patients with CD40 deficiency display a more severe clinical phenotype. It has been previously shown that CD40L is not the only ligand that can bind to CD40. A protein that binds activated complement 3b (C3b) and C4b, C4BP, on FDCs can also deliver signals to B cells *via* CD40 during T-cell independent responses (26). Possibly, the lack of these T-independent signals in human CD40 deficiency might have contributed in the aggravated clinical phenotype of this form of HIGM.

Antigen-specific interactions between Tfh cells and B cells are required from the very first stage of their interaction to allow B-cell differentiation toward the GC fate and the maintenance of the GC response (27). B cells in return, provide signals for GC Tfh formation to Tfh cells suggesting that B cells and Tfh cells are mutually dependent on each other for their differentiation into GC B cells and GC Tfh cells, respectively (17–19, 28, 29). CD40L–CD40 interactions along with integrin and SAP-dependent contacts between B and T cells were shown to be essential for this process (30–32). In agreement to previous reports, we found that patients with CD40L deficiency generated a small number of Tfh cells (21). Instead, circulating Tfh cells in a patient with CD40 deficiency were heavily compromised. Although the blood is not the most suitable tissue to address the effect of CD40:CD40L signaling in Tfh development, our results suggest that other mechanisms might be able to compensate for the lack of CD40L. *AICDA* mutations on the other hand, increased the number of circulating Tfh cells in line with previous results supporting the notion that the reduced efficiency in generating highly mutated antibodies leads to enhanced GC reactions and increased Tfh cell development (16).

Both CD40L and CD40 deficiencies were characterized by normal or even elevated concentration of CXCL13 in the plasma. Tfh along with FDCs are the main source of CXCL13 in the B cell follicles (23). Recently, a CXCR5^−^PD-1^++^ Tfh population was described in the tumor infiltrate of patients with breast cancer and the synovial fluid of patients with rheumatoid arthritis as an important source of CXCL13 (33). The percentage of CXCR5^−^PD-1^++^ cells was not increased in the blood of CD40- nor CD40L-deficient patients (data not included), suggesting the FDCs were the main source of CXCL13. Alternatively, the lack of “CXCL3 consumption” by GC Tfh and B cells might have led to an increase in the plasma CXCL13 levels in human CD40 and CD40L deficiency.

## Concluding Remarks

Although studies on additional CD40-deficient patients are necessary, analysis of an individual with CD40 deficiency suggests that CD40 is possibly required for Tfh and Tfr development in humans. Compared with other genetically characterized forms of HIGM, i.e., due to mutations in *CD40L* and *AICDA*, patients with CD40 deficiency show a more severe clinical phenotype. According to our analyses, this could be partially explained by the more severe impairment of Tfh and Tfr cells. Longitudinal studies on a larger number of samples, i.e. blood and secondary lymphoid organs from patients with CD40 deficiency may offer useful insights in human Tfh biology, with important implications for understanding human GC development and potentially for the management of patients affected with HIGM or other primary immunodeficiencies.

## Ethics Statement

All the subjects, their parents, or tutors for minors gave their written informed consent. The study was approved by the local ethic committee (Comitato Etico Ospedale San Raffaele, Milano) and performed in accordance with the Declaration of Helsinki.

## Author Contributions

MC: contributed to scientific discussion and wrote the manuscript. JG: performed the experiments and data analysis and contributed to manuscript writing. MB: provided samples and contributed to manuscript writing. DM, FL, AS, RD, MM, LB, SV, CD, AP, and AA: provided samples and read and approved the manuscript. VL: provided samples, contributed to scientific discussion, and wrote the manuscript. GF: designed and supervised the study, coordinated scientific discussion and wrote the manuscript.

## Conflict of Interest Statement

The authors declare that the research was conducted in the absence of any commercial or financial relationships that could be construed as a potential conflict of interest.

## Supplementary Material

Figure S1 Geometric mean fluorescence intensity (gMFI) of PD-1 in a patient with CD40 deficiency compared with patients with *CD40L* and *AICDA* mutations. **(A–C)** gMFI of PD-1 on Tfh **(A)**, Tfr **(B)** and Treg cells **(C)** in pediatric and adult hyper-IgM (HIGM) patients compared with age-matched health controls (HCs) (same as in Figure 1). Bars: mean ± SEM. Each dot represents one patient. Black & white squares represent longitudinal measurements of the same CD40 ligand (CD40L)-deficient patient, collected at 14 and 19 years of age.

## Abbreviations

HIGM, hyper-IgM; CSR, class switch recombination; SHM, somatic hypermutation; GC, germinal center; AID, activation-induced cytidine deaminase; CD40L, CD40 ligand; UNG, uracil *N*-glycosylase; HC, healthy control; Tfh, T follicular helper; Treg, T regulatory; Tfr, T follicular regulatory; FDCs, follicular dendritic cells; C3b, complement 3b; TMP–SMZ, trimethoprim/sulfamethoxazole; PCP, *Pneumocystis carinii* pneumonia; HSCT, hematopoietic stem cell transplantation.
