# Supplementary figures and images for: Circulating Follicular Helper and Follicular Regulatory T Cells Are Severely Compromised in Human CD40 Deficiency: A Case Report
Source: Front Immunol. 2018 Aug 6;9:1761. doi: 10.3389/fimmu.2018.01761 (PMC6090258; doi:10.3389/fimmu.2018.01761)

Supplementary Figure 1

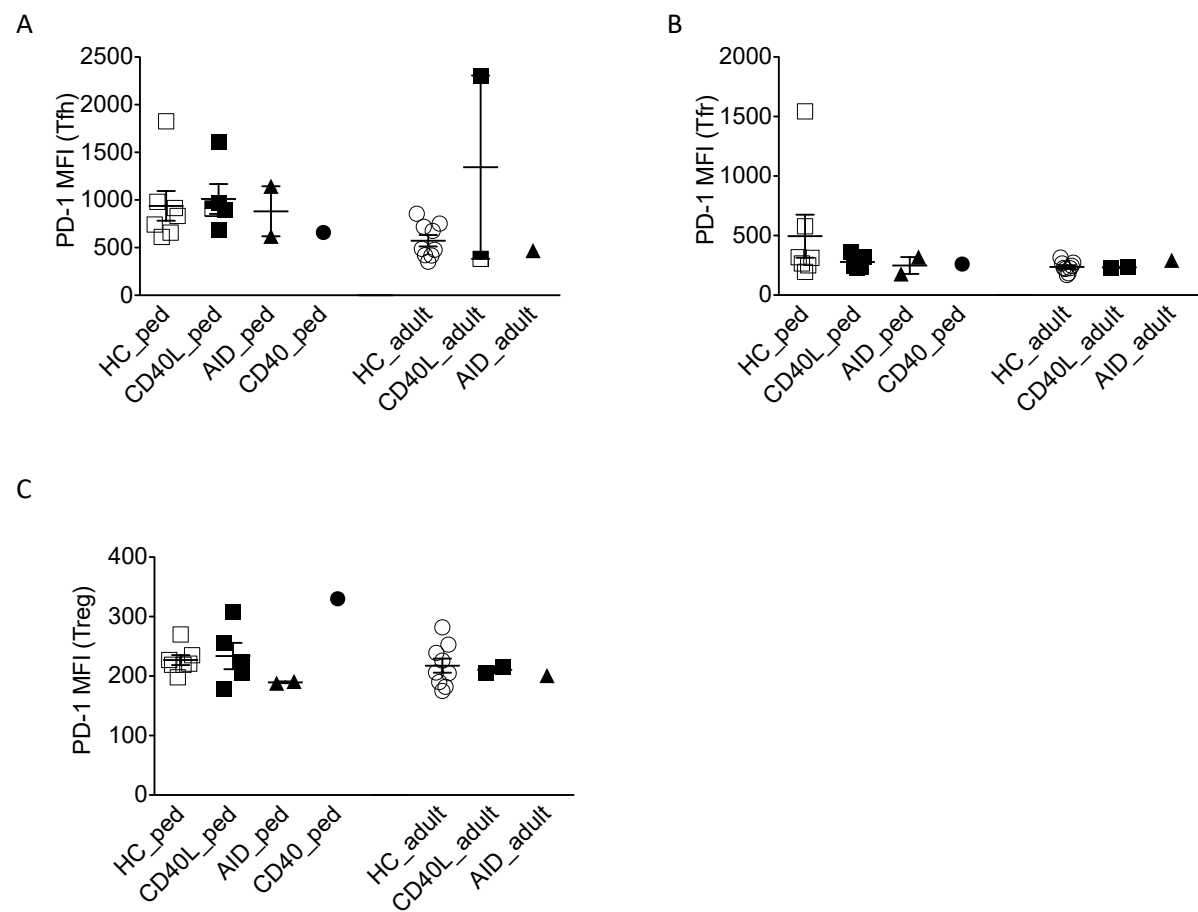

Supplement: Figure S1 — Geometric mean fluorescence intensity (gMFI) of PD-1 in a patient with CD40 deficiency compared with patients with CD40L and AICDA mutations. (A–C) gMFI of PD-1 on Tfh (A), Tfr (B) and Treg cells (C) in pediatric and adult hyper-IgM (HIGM) patients compared with age-matched health controls (HCs) (same as in Figure 1). Bars: mean ± SEM. Each dot represents one patient. Black & white squares represent longitudinal measurements of the same CD40 ligand (CD40L)-deficient patient, collected at 14 and 19 years of age. [file image_1.pdf]
